# Parasitism Affects Entomofauna Dynamics in Infected and Uninfected Plants: A Case Study of *Orobanche anatolica* Parasitizing *Salvia absconditiflora*

**DOI:** 10.3390/insects15120929

**Published:** 2024-11-27

**Authors:** Çiğdem Özenirler

**Affiliations:** Applied Biology Section, Department of Biology, Faculty of Science, Hacettepe University, 06800 Ankara, Türkiye; cozenir@hacettepe.edu.tr; Tel.: +90-3122978055

**Keywords:** entomofauna, *Salvia absconditiflora*, biodiversity, parasitism, nectar

## Abstract

*Salvia absconditiflora* and *Orobanche anatolica* exhibit a parasitic relationship. *S. absconditiflora* serves as a rich food resource for insects due to its abundant nectar. *O. anatolica* demonstrates host specificity towards *S. absconditiflora*. The objective of this study is to assess potential variations in entomofauna dynamics and nectar chemistry attributable to parasitism. The observational groups consisted of *S. absconditiflora* individuals infected and uninfected by *O. anatolica*, as well as *O. anatolica* individuals. Entomofauna was monitored during the flowering periods of target species. The collected data were analyzed using biodiversity indexes. The nectar of the plant groups was evaluated with LC-QTOF-MS analysis to identify possible repellents for insects. *S. absconditiflora* plants free from infection had significantly higher insect diversity and abundance compared to those infected by *O. anatolica*. Parasitism by *O. anatolica* appeared to modify the entomofauna dynamics of *S. absconditiflora*, as demonstrated by the disparities in insect diversity metrics between infected and uninfected plants. When comparing the nectars of the plant groups, it was determined that the nectar from the parasitic plant and the infected *S. absconditiflora* contained substances with repellent properties in higher quantities than that of uninfected *S. absconditiflora*, including thymol, n-benzyloleamide, azadirachtin, capsaicin, azatadine maleate, and andrachcinidine. This study concluded that parasitism could lead to differences in entomofauna associated with these species.

## Simple Summary

This study examines the relationship between a parasitic plant and its host, focusing on how nectar composition affects insect visitors. It was found that an infected plant attracted fewer insects compared to its uninfected counterparts, likely due to less appealing nectar. Its nectar contained various compounds that might repel insects, suggesting a defense mechanism. The findings indicate that while some parasitic plants do not rely on pollinators, they still produce abundant nectar, mainly consumed by ants. This research is significant as it reveals how these plants may affect their host’s growth and nutrient uptake, potentially offering insights into the development of natural insect repellents. Such plant-based solutions could benefit agriculture and reduce reliance on chemical pesticides, promoting a healthier environment.

## 1. Introduction

*Salvia absconditiflora* Greuter & Burdet (Lamiaceae) (synonym—*Salvia cryptantha* Montbret & Aucher ex Bentham) is an endemic species in Turkey and serves as a rich food resource for insects due to its abundant nectar and pollen [1]. The genus *Orobanche* L. from the Orobanchaceae family consists of obligate root parasites devoid of chlorophyll in their aboveground organs [2,3]. These taxa are among the most agronomically destructive parasitic plants globally [4]. *O. anatolica* Boiss. & Reut. shows host specificity behavior on *S. absconditiflora*, while other species can have a broad potential host range [5].

The zygomorphic, two-lipped flowers of *Orobanche* (broomrape) attract insects with their significant fragrance [6] and offer pollen and nectar rewards [7]. The flowers of these broomrapes are adapted to insect pollination, although they are capable of self-pollination as well [8,9]. Nectar is one of the most important keystones connecting angiosperms and nectar-feeding insects [10,11]. Nectar is the main floral reward provided by the majority of angiosperms [12]. The coevolution of plants and their pollinators stands out as a key mechanism shaping angiosperm evolution [13,14]. Despite this, our comprehension of the evolution of floral nectar remains limited. In the course of evolution, plants have developed different strategies to attract or repel other living organisms. A deeper understanding of how a plant can employ attractants and/or deterrents can significantly contribute to unraveling evolutionary perspectives on ecosystem working conditions [15].

Nectar varies according to the plant species and habitat, while simultaneously serving as a crucial food source for many organisms due to its instant nutrient availability. Flowering plants often produce hundreds of different floral scents [16,17,18]. These phytochemicals are an adaptation mechanism that plants have evolved to attract/repel and guide insects [19,20,21].

Floral nectar is one of the most important keystones connecting angiosperms and pollinators. It is a sugary liquid produced by nectaries and composed of water, sugars, amino acids, lipids, vitamins and minerals, phenolic compounds, organic acids, secondary metabolites, proteins, and enzymes. Secondary metabolites include alkaloids and glycosides. These compounds can serve as defense mechanisms against herbivores and pathogens. The precise composition of floral nectar can vary widely among different plant species, and even within species depending on environmental conditions and plant health.

This study investigates the obligatory parasitic relationship between *S. absconditiflora* and *O. anatolica*, with a focus on the potential differences in nectar composition between infected and non-infected individuals. It aims to explore how these nectar variations relate to the diversity of insect visitors, thereby determining the impacts of *O. anatolica* parasitism on the entomofauna dynamics and nectar chemical composition of *S. absconditiflora*. The observational groups included *S. absconditiflora* individuals infected and uninfected by *O. anatolica*, as well as individuals of *O. anatolica* itself.

## 2. Materials and Methods

Study area: The study area is located within Hacettepe University Beytepe Campus (Ankara, Turkey) situated at approximately 1000 m above sea level (39°52′10″ N 32°44′06″ E). This area is characterized by its steppe vegetation. The target plants were classified into three groups based on their parasitic status. Group 1 includes non-infected *S. absconditiflora* individuals, Group 2 consists of infected *S. absconditiflora* individuals, and Group 3 includes *O. anatolica* individuals that live parasitically on *S. absconditiflora*. In this framework of classification, the insects visiting the mentioned plant groups were also categorized into Groups 1, 2, and 3. Similarly, the nectar samples collected from the plants within these groups were also categorized into Groups 1, 2, and 3.

Observations on pollinator/visitor insects: Observations and sampling of insects collecting nectar and/or pollen on target plants were carried out over six days in the blooming periods of the plant groups. The study commenced when the flowering of the plants reached 100%. Sampling was conducted over a six-day period from 24 May to 1 June 2022, during which weather conditions were dry with no rainfall. Sampling was conducted daily at six time points—07:00, 09:00, 11:00, 14:00, 16:00, and 18:00—with each sample collected during a 10 min interval. In total, 108 samples were collected. For each group, 15 individuals were observed.

Identification of the insects: All materials were caught by aerial nets after their contact with target flowers. The samples were preserved as museum material in İstanbul University’s Zoology Collection. Taxa identifications were made using various sources from the literature [22,23]. Insect identifications were confirmed by F. Dikmen, University of İstanbul.

Determination of the alkaloids in nectar: Nectar collection involved selecting 15 individuals from each group. Nectar samples were collected daily for Group 1 (15 individuals × 5 flowers), Group 2 (15 individuals × 5 flowers), and Group 3 (15 individuals × 5 flowers). This sampling was conducted over a period of six days during which insect observations were carried out. Nectar collection was performed using sterile microhematocrit tubes (ISO 12772, REF 160260, Vitrex Medical A/S, Herlev, Denmark), and the samples were transported to the laboratory on ice and stored at −80 °C until analysis. The nectar collected for each sample group was analyzed by pooling a minimum volume of 3 mL.

LC-QTOF-MS metabolomic analysis was performed to identify and quantify the metabolites present in each collected nectar sample. Each collected nectar sample was prepared by dissolving 5 μL of water–acetonitrile (50:50, *v*/*v*) mixture, and the samples were analyzed by optimized LC-QTOF-MS (Agilent 6530, Agilent Technologies, Santa Clara, CA, USA) using a C18 (2.1 × 100 mm, 2.7 μm) column with moving phases of water containing 0.1% formic acid (A) and acetonitrile containing 0.1% formic acid (B). The flow rate was 0.3 mL/min, and the injection volume was 10 μL. The complex chromatograms obtained were separated using MS-Dial [24] software, and data matrices were created by correcting the retention times of the peaks. MS/MS fragmentation of plant metabolites was performed by applying a collision energy of 20 eV to reliably identify peaks in the data matrix. The minimum peak height for peak detection was set to 2000, and MS1 and MS2 tolerances were set to 0.01 and 0.025 daltons. The raw data of this study were processed with MetaboAnalyst [25,26]. Metabolite enrichment analysis was used to reveal the 25 most altered metabolites based on differentially expressed metabolites identified in Group 1, Group 2, and Group 3.

Statistical analysis: Flower-visiting insects among the sample groups were compared using biodiversity indices according to Krebs (1999) and Magurran (2004) [27,28]. To evaluate biodiversity among the three plant groups, several ecological indices were calculated in R [29] to obtain the summary results of each sampling group. The ecological indices used in this study are as follows: dominance (*D*) to evaluate species dominance; Simpson (1-*D*) and Shannon (*H*) to measure diversity and richness; Evenness_e^H/S to assess species evenness; Brillouin to quantify diversity accounting for sample size; Menhinick and Margalef to estimate species richness relative to sample size; Equitability (*J*) to gauge evenness in species distribution; Fisher’s alpha to estimate total species richness; Berger–Parker to identify the dominance of the most abundant species; and Chao1 to estimate the number of undetected species.

## 3. Results

### 3.1. Insects

**Figure 1:**
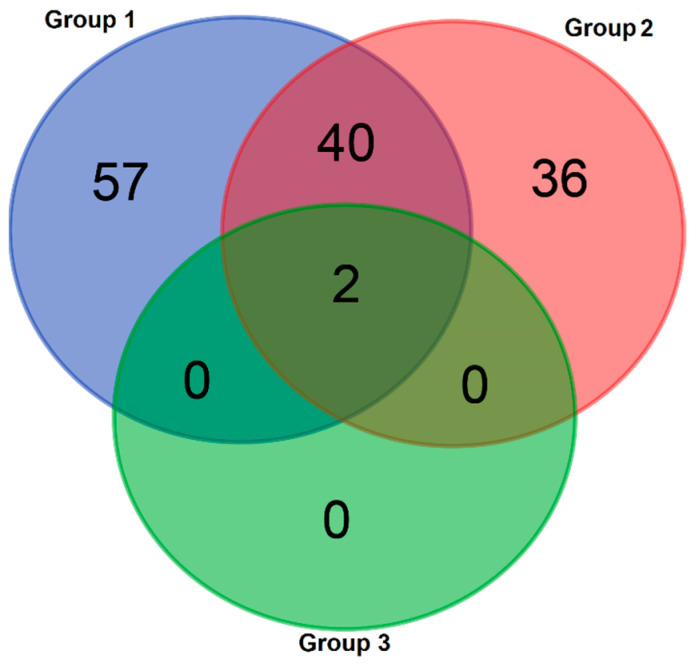
Venn diagram illustrating the distribution of insect taxa among three plant groups: Group 1 (insects visiting *S. absconditiflora* individuals not infected by *O. anatolica*), Group 2 (insects visiting *S. absconditiflora* individuals infected by *O. anatolica*), and Group 3 (insects visiting *O. anatolica* individuals).

A total of 297 individuals from 95 species visiting Group 1 and 161 individuals from 75 species visiting Group 2 were identified. A total of 32 individuals from two species (Formicidae sp1 and sp3) visiting only Group 3 were recorded. The results were simply summarized with a Venn diagram (Figure 1). Group 1 includes 57 unique taxa (Coleoptera (Carabidae sp1, Curculionidae sp1, *Mordella* sp1, Mordellidae sp1, Scarabaeidae sp1, Scarabaeidae sp4, Coleoptera sp4, sp5, sp6, sp9, sp10, sp11, sp12), Diptera (Bombylidae sp1, Diptera sp4, sp5, sp6, sp7), Hemiptera (*Corizus hyoscyami*, Hemiptera sp1, sp3, sp4, sp5, sp7, Cicadellidae sp1, sp3, Miridae sp1, *Orthotylus* sp1), Hymenoptera (*Apis mellifera*, *Bombus terrestris* (worker), *Bombus terrestris* (queen), *Bombus niveatus* (worker), *Bombus niveatus* (queen), *Andrena* sp2, Andrenidae sp4, *Ceratina* sp2, *Eucera* sp7, sp8, *Nomada* sp1, *Xylocopa violacea*, *Chelonus* sp1, Chrysididae sp1, *Crabro* sp1, Formicidae sp2, sp4, *Lasioglossum* sp2, sp6, Ichneumonidaen sp2, *Anthidium* sp1, *Megachile* sp1, sp6, Megachilidae sp11, sp12, *Tenthredo* sp1), Lepidoptera sp1, and Orthoptera (Tettigoniidae sp1, sp3)). Group 2 contains 36 unique taxa (Coleoptera (*Anthaxia* sp1, *Scymnus interruptus*, Dermestidae sp1, Coleoptera sp2), Diptera (Bombylidae sp2, Diptera sp1), Hemiptera sp6, Hymenoptera (*Apis mellifera*, *Andrena* sp1, sp3, Andrenidae sp5, Anthophora sp2, sp3, *Anthophora plumipes*, *Ceratina* sp1, sp3, *Eucera* sp2, sp3, sp6, sp9, Apidae sp1, Braconidae sp3, *Lasioglossum* sp10, sp3, sp8, sp9, *Halictus resurgens*, Ichneumonidae sp3, *Megachile* sp2, Megachilidae sp3, sp8, Vespoidae sp1, sp2), and Orthoptera (Acrididae sp2, Tettigoniidae sp2, sp5)).

The intersection between Group 1 and Group 2 encompasses 40 taxa (Coleoptera (Bruchidae sp1, *Polydrusus* sp1, *Oxythyrea funesta*, *Phyllopertha horticola*, Coleoptera sp1, sp3, sp7), Diptera (*Ceroxys* sp1, Tephritidae sp3, Diptera sp2, sp3), Hemiptera (*Cercopis vulnerata, Macrosteles* sp1), Hymenoptera (*Anthophora plumipes*, *Anthophora* sp1, sp4, sp5, *Apis mellifera*, *Bombus niveatus*, *Eucera* sp1, sp4, sp5, *Lasioglossum marginatum*, *Lasioglossum* sp1, sp4, *Anthidium* sp2, Megachilidae sp4, sp5, sp6, sp7, sp9, Tiphiidae sp1, sp2, *Polistes dominula*), Lepidoptera (*Coleophora argentula*, Sphingidae sp1, *Cydia* sp1), and Orthoptera (Acrididae sp1, sp3, Tettigoniidae sp4)), suggesting that a substantial portion of insect visitors are shared between the infected and non-infected *S. absconditiflora* individuals. However, the intersection of all three groups is limited to just two taxa, indicating that while there is some overlap between insects visiting *S. absconditiflora* and *O. anatolica*, these taxa are not specific to *O. anatolica* individuals alone. This analysis underscores a clear separation in insect communities between the plant species and highlights a unique subset of taxa that interact with both forms of *S. absconditiflora*, but not with *O. anatolica*.

Group 1 was found to be predominantly represented by Hymenoptera (66.33%), followed by Coleoptera (12.12%), Hemiptera (11.44%), Diptera (4.72%), and Lepidoptera (2.02%). For Group 2, the predominant representative was Hymenoptera (57.14%), followed by Hemiptera (15.75%), Coleoptera (11.80%), Diptera (7.46%), Orthoptera (5.59%), and Lepidoptera (1.86%). Group 3 was exclusively represented by Hymenoptera (100%), with only Formicidae members identified.

The following are members of the Hymenoptera order that visited both Groups 1 and 2: *Andrena* spp. (5 species) from the family Andrenidae; *Apis mellifera*, *Bombus terrestris*, *Bombus niveatus* (queen), *Bombus argillaceus* (queen), *Ceratina* spp. (3 species), *Xylocopa violacea*, *Eucera* spp. (9 species), *Nomada* sp., *Anthophora* spp. (4 species), and *Anthophora plumipes* from the Apidae family; *Lasioglossum* spp. (10 species), *Lasioglossum marginatum*, and *Halictus resurgens* from the family Halictidae; 12 species from the family Megachilidae; and from the parasitoid and wasp group, Braconidae (2 spp.), *Chelonus* sp., Chrysididae sp., Crabronidae (*Crabro* sp.), Formicidae (4 spp.), Ichneumonidae (3 spp.), Tenthredinidae (*Tenthredo* sp.), Vespidae (*Polistes dominula*), and 2 other wasp species. In total, 68 taxa were identified.

**Figure 2:**
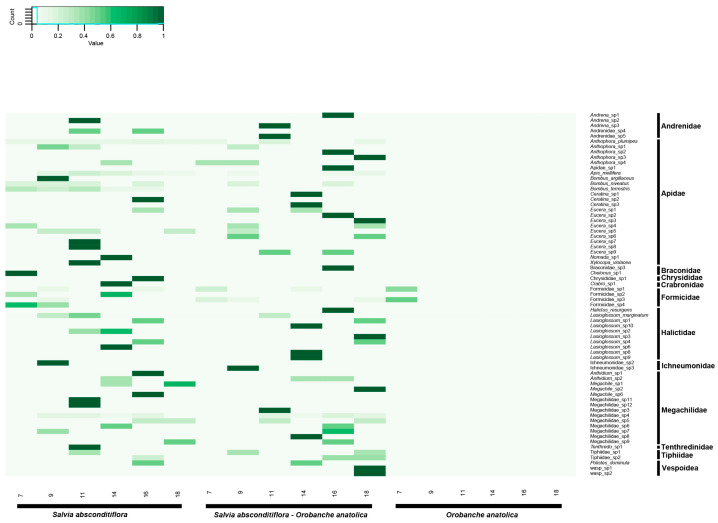
Heat map displaying the distribution of Hymenoptera taxa across three groups (Group 1—*S. absconditiflora*; Group 2—*S. absconditiflora* individuals infected by *O. anatolica*; and Group 3—*O. anatolica*) at different times of the day (07:00, 09:00, 11:00, 14:00, 16:00, and 18:00). The *X*-axis indicates sampling times, while the *Y*-axis represents Hymenoptera groups.

The taxa of insects classified within the Hymenoptera order were summarized using heat map graphics based on plant groups and the six sampling times throughout the days (Figure 2). In this graph, the first column represents Group 1, the second column represents Group 2, and the third column represents Group 3. On the *X*-axis are the sampling times during the day (07:00, 09:00, 11:00, 14:00, 16:00, and 18:00), and on the *Y*-axis are the Hymenoptera groups.

The activity of Hymenoptera in Group 3 was detected exclusively during observations made at 7:00 a.m. Both taxa identified were members of the Formicidae family. The activity of Andrenidae members in Group 1 was generally observed to begin around 11:00 a.m. and continued into the afternoon. Members of the Apidae family exhibited relatively uniform activity throughout the day. Additionally, *Bombus niveatus* and *Bombus argilleceus* queens emerging from hibernation were observed during the early hours of the day. The activity of relatively smaller-bodied bees, such as Halictidae members, showed a pronounced increase in the afternoon. Furthermore, the overall activity of Megachilidae members was higher between 11:00 a.m. and 4:00 p.m.

Evaluations of dominance were conducted on visiting insects within the framework of the Berger–Parker dominance index. In Group 1, *Apis mellifera* (Hymenoptera) dominates with 31.31%, followed by *Bombus terrestris* (Hymenoptera) (7.40%), *Cercopis vulnerata* (Hemiptera) (6.73%), and *Polydrusus* sp1 (Coleoptera) (3.03%). In Group 2, *Cercopis vulnerata* (Hemiptera) dominates with 14.90%, and other dominant taxa include *Apis mellifera* (Hymenoptera) (8.07%), Megachilidae sp4 (Hymenoptera) (6.83%), *Polydrusus* sp1 (Coleoptera) (3.72%), and *Anthophora plumipes* (Hymenoptera) (3.10%).

The biodiversity metrics for the Group 1, Group 2, and Group 3 datasets reveal substantial differences in ecological diversity (Table 1). The Group 1 dataset exhibits a species richness (Taxa_S) of 95 and a high number of individuals (297), alongside a relatively low dominance index (Dominance_D = 0.1139), suggesting a relatively balanced community structure. This is further supported by high values on Shannon’s diversity index (Shannon_H = 3.387) and Simpson’s 1-D index (0.8861), reflecting considerable diversity. The evenness of species distribution is lower in Group 1 (Evenness_e^H/S = 0.3112), indicating some level of dominance by certain species. The Brillouin index (3.014) and Margalef’s richness index (16.51) corroborate this, suggesting a diverse community with moderate evenness. In contrast, Group 2 shows slightly reduced species richness (Taxa_S = 75) and fewer individuals (161), but still maintains a low dominance index (Dominance_D = 0.04325) and high diversity indices (Shannon_H = 3.803; Simpson_1-D = 0.9568), indicating high ecological diversity. Evenness (Evenness_e^H/S = 0.5981) is higher than in Group 1, though the Brillouin index (3.273) and Margalef’s index (14.56) reflect a somewhat less diverse community relative to Group 1. In stark contrast, Group 3 presents a very low species richness (Taxa_S = 2) and a minimal number of individuals (32), accompanied by a high dominance index (Dominance_D = 0.5488). The low Shannon’s index (Shannon_H = 0.6435) and Simpson’s 1-D index (0.4512) indicate significantly reduced diversity. Despite high evenness (Evenness_e^H/S = 0.9516), the community’s low species richness and high Berger–Parker index (0.6563) suggest that a few species dominate the limited ecological space. The Chao-1 estimator (2) further underscores the minimal species diversity in Group 3, indicating that the dataset represents a simple community structure.

### 3.2. Metabolomics

**Figure 3:**
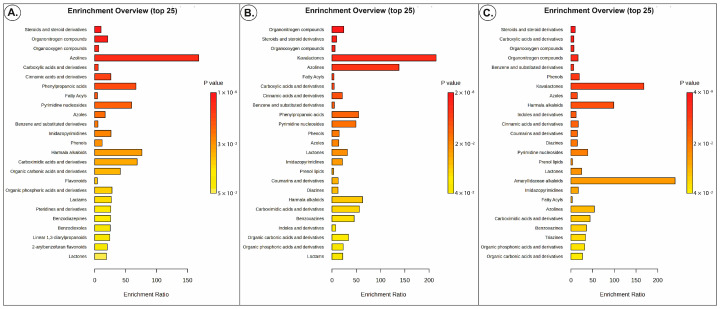
Results of metabolite set enrichment analysis (MESA) based on quantitative metabolomic data of Group 1 (**A**), Group 2 (**B**), and Group 3 (**C**) nectar samples. Bar lengths are based on the enrichment ratio. Color intensity (white to red) reflects increasing statistical significance; *y* axis—*p*-values from metabolite enrichment analysis; *x* axis—impact values derived from topology analysis. Group 1—non-infected *Salvia absconditiflora*; Group 2—*S. absconditiflora* infected with *Orobanche anatolica*; Group 3—*O. anatolica*.

According to the positive ion results of the nectars, 2203 peaks were detected, while the negative ion results showed 61 peaks. Initially, the data of 2203 metabolites were cleaned of duplicates, resulting in the identification of 589 metabolites (Table S1). Metabolite set enrichment analysis showed the 25 most altered metabolites as revealed based on differentially expressed metabolites identified in all nectar sample groups. The graph was obtained by plotting the -log of *p*-values from metabolite enrichment analysis on the *y*-axis and the pathway impact values derived from pathway topology analysis on the *x*-axis. Bar colors are based on *p*-values (lower *p*-values correspond to darker red), while bar lengths are based on the enrichment ratio. Color intensity (yellow to red) reflects increasing statistical significance (Figure 3). Upon analyzing the constituents of the three groups, significant variations were observed in both the amounts and profiles of these constituents among the samples. The nectar profile of *O. anatolica* appears to be remarkably rich compared to that of *S. absconditiflora*. The field observations align with the findings, indicating that *O. anatolica* produces more abundant nectar than *S. absconditiflora*. The phytochemical results reveal that all nectar samples contain significant amounts of carbohydrates, amino acids, phenolic substances, and alkaloids.

## 4. Discussion

Understanding the intricate relationship between plants and insect visitors provides valuable insights into the evolutionary process. In this context, the observations of *O. anatolica* reveal a notable outcome: the parasitic plant exhibits almost no insect visitors, and further analysis indicates that *S. absconditiflora* plants infected with *O. anatolica* attract fewer insect visitors than their uninfected counterparts. A total of 57 taxa from seven distinct orders were identified visiting Group 1, while Group 2 hosted 51 taxa from seven orders. Group 3, on the other hand, was exclusively visited by Hymenoptera (100%), with only two different Formicidae members. This difference suggests that the roles of host and parasitic plant nectars in the evolutionary process may differ within this context. Ollerton et al. 2007 evaluated the relationship between a parasitic plant and a host according to different strategies. They showed that the nectar concentration and number of visitors of *O*. *elatior* are lower than the host [7]. According to Bani et al. (2018), *Orobanche nowackiana* exerts a detrimental effect on the growth and nutrient dynamics of *Artemisia murale*. The parasitic invasion leads to a significant reduction in key growth metrics of *A*. *murale*, including dry weight, shoot length, root length, and branch number. Moreover, *O*. *nowackiana* diminishes mineral nutrient concentrations in the host, particularly phosphorus (P) and potassium (K). The elevated levels of these nutrients in *O*. *nowackiana* compared to the host suggest a selective uptake mechanism, underscoring the parasite’s impact on the host’s nutrient allocation and overall vitality [30]. Although this was not investigated in this study, because of the parasitic relationship between *S. absconditiflora* and *O. anatolica*, it is possible to see that infected individuals’ nectar content is less attractive to the insects than that of the uninfected individuals. The *Orobanche* genus is known for its rapid growth and short flowering time [31]. Early anthesis time and self-fertilization could be other strategies adaptive to parasitic life and for ensuring survival. Therefore, pollinators are not necessary for some of the *Orobanche* species. The results regarding the insect visitors of *O. anatolica* support this conclusion. For *Orobanche*, the primary goal is to complete their life cycle and spread “dust seeds” as close to the host plant as possible, ensuring germination [32,33]. Researchers posit that the size and number of seeds per capsule may be the result of their attempt to increase their chance of finding a host [34,35]. Therefore, to increase their chances of germination, these plants produce large quantities of very small seeds, and the proximity of these seeds to the roots greatly increases their chances of finding a host. The appearance of ants on *O. anatolica* may be related to seed dispersal rather than pollination. Through careful observations, it has been determined that *O. anatolica* nectar is notably abundant. Interestingly, this plentiful nectar is preferred exclusively by Formicidae individuals, with no consumption observed among other insects in the area. The prevailing hypothesis suggests that this nectar’s abundance is linked to the parasitic plant’s ability to derive all necessary nutrients from its host. The transpiration pressure from the host to the parasite facilitates the transfer of water and nutrients. The development of shoots and germination in *Orobanche* is closely tied to the nutritional status and soil temperature of both the parasite and its host plant. However, a complete understanding of the extent of phloem interaction between the parasite and the host has still not been fully achieved. In the short life cycle of *Orobanche*, it is likely that they protect themselves from herbivore insects through the help of some chemicals in nectar. Previous studies indicated that the nectar profiles of *Orobanche* species show variation in their composition [36]. El-Akkad et al. (2002) and Hassan and El-Awadi (2009) detected the presence of lipid, lignin, phenolic, and suberin materials [37,38]; Sacchetti et al. (2003) described the content of terpenes and flavonoids [39]; Serafini et al. (1995) demonstrated the presence of phenylpropanoid glycosides [40]; and Tóth et al. (2016) detected floral volatile organic compounds [18]. On the other hand, a group of these components including alkaloids [41,42,43,44], polyphenols [45], coumarins, saponins, and non-protein amino acids can make nectar toxic for some animals or repellent to insects [43,46,47]. As insect repellents or/and insecticides, thymol, n-benzyloleamide, azadirachtin, capsaicin, azatadine maleate, and andrachcinidine were detected in Group 3. Among these, thymol, isolated from *Thymus vulgaris* (Lamiaceae), is an active compound against a variety of insects [48]. Andrachcinidine is a piperidine-containing alkaloid, recorded as a chemical defense agent of the insect *Andrachne aspera* [49]. This substance, which may be related to insect visits, may also be related to the insects that are distributed in our study area and may be a subject for future studies. Azadirachtin is a triterpenoid compound isolated from *Azadirachta indica*, a biological insecticide which impairs the growth and molting process of Hemiptera, Lepidoptera, Coleoptera, and other pests [50,51]. It can cause damage to the midgut epithelial cells of foraging insects [52]. The results of this study indicate that nectar from non-infected *S. absconditiflora* is preferentially chosen by members of the Coleoptera and Hemiptera taxa present in the area. Capsaicin is obtained by grinding dried, ripe *Capsicum* spp. (chili peppers) into a fine powder. Capsaicin is used as an insect pest repellent and insecticide [53]. As shown in Figure 3, in Group 3, harmala alkaloids were found much more than in Group 1 and Group 2. Amarylidacea alkaloids were only found in Group 3 nectar. Phytochemicals found in Group 3 in high amounts were alkaloids, which included prolintane triethyl phosphate, theobromine, trigonelline hydrochloride, berberine chloride, conessine, the β-carboline class of alkaloids (norharman harmaline, harman) and the pyrolizidine alkaloid (anabasamine). All of these alkaloids are known for different activities, such as their repellent effects or effects on the nervous systems of animals, or for their psychoactive properties [54]. Dicyclohexylurea, aminopyrine, and azodiractin and its derivatives have also been studied for their potential insecticidal properties [51,55]. Azodiractin acts as an antifeedant, repellent, and growth regulator, disrupting the life cycle of insects. It affects insect behavior, feeding, molting, and reproduction. DIBOA is a member of the benzoxazinoid class of compounds. These secondary metabolites are produced by plants as a defense mechanism against herbivores, pathogens, and other environmental stresses. It can act as a feeding deterrent and has toxic effects on certain insects and other pests. Additionally, DIBOA and related compounds have been investigated for their potential to improve plant health and resistance to stress [56,57,58,59]. Our findings not only show why *O. anatolica* nectar is not attractive to insects but also are very important in terms of identifying a new source plant for the production of a plant-based repellent or insecticide. Natural, plant-based insect repellents or insecticides have become more widely used because they are soluble, environmentally friendly, and relatively less harmful for the environment [60]. These compounds generally provide an advantage to plant populations by stopping the life cycles of insects, causing insects not to choose the plant as food, or causing them not to choose the plant as a micro-habitat.

## 5. Conclusions

Based on the study results, the differences in insect visitation and secondary metabolites in the nectar of *S. absconditiflora* individuals infected and not infected with *O. anatolica* are elucidated. The analysis revealed that the nectar of the parasitic plant contains higher concentrations of compounds with repellent or insecticidal properties. Despite the diversity of insect species in the area, it was observed that only two ant species visited this group of plants. In light of this information, it is suggested that the potential of *O. anatolica* as a source of plant-based repellents or insecticides merits further investigation.

## Figures and Tables

**Table 1:** Biodiversity metrics of visitor insects. Group 1 (insects visiting *S. absconditiflora* individuals not infected by *O. anatolica*), Group 2 (insects visiting *S. absconditiflora* individuals infected by *O. anatolica*), and Group 3 (insects visiting *O. anatolica* individuals).

	Group 1	Group 2	Group 3
Taxa_S	95	75	2
Individuals	297	161	32
Dominance_D	0.1139	0.04325	0.5488
Simpson_1-D	0.8861	0.9568	0.4512
Shannon_H	3.387	3.803	0.6435
Evenness_e^H/S	0.3112	0.5981	0.9516
Brillouin	3.014	3.273	0.5836
Menhinick	5.512	5.911	0.3536
Margalef	16.51	14.56	0.2885
Equitability_J	0.7437	0.8809	0.9284
Fisher_alpha	48.3	54.62	0.4729
Berger–Parker	0.3131	0.1491	0.6563
Chao-1	303	141.4	2

## Data Availability

All data have been shared within the manuscript and as Supplementary Data.

## Supplementary Materials

The following supporting information can be downloaded at https://www.mdpi.com/article/10.3390/insects15120929/s1, Table S1: The results of nectar metabolomic analyses.
